# Enteric parasitic infection among HIV-infected patients visiting Tribhuvan University Teaching Hospital, Nepal

**DOI:** 10.1186/s13104-016-2007-5

**Published:** 2016-04-06

**Authors:** Ananda Ghimire, Shiva Bhandari, Sarmila Tandukar, Jyoti Amatya, Dinesh Bhandari, Jeevan Bahadur Sherchand

**Affiliations:** Blood Transfusion Service, Department of Pathology, Tribhuvan University Teaching Hospital, Maharajgunj, Kathmandu, Nepal; Nutrition Innovation Lab, Nepal/Johns Hopkins University, Patan Dhoka, Lalitpur, Kathmandu, Nepal; Public Health Research Laboratory, Institute of Medicine, Tribhuvan University Teaching Hospital, Maharajgunj, Kathmandu, Nepal; Department of Microbiology, Trichandra Multiple College, Ghantaghar, Kathmandu, Nepal

**Keywords:** CD4 T-cell, Diarrhea, Enteric parasites, HIV, Multi-parasitism

## Abstract

**Background:**

Enteric parasitic infection among human immunodeficiency virus (HIV) patients has been a significant health problem in developing countries like Nepal. This study was undertaken to access the burden of enteric parasites among HIV patients and its association with their immune status.

**Methods:**

A cross-sectional study, involving 112 HIV sero-positive patients was conducted in Tribhuvan University Teaching Hospital, Public Health Research Laboratory, Kathmandu, Nepal from July 2011 to June 2012. The fecal samples were processed by direct-smear technique, in both normal saline solution and 1 % iodine solution as well as modified acid fast staining (Kinyoun’s method) after formalin ether concentration and Sheather’s sucrose flotation for the identification of enteric parasites.

**Results:**

Infection with one or more parasite was seen in 33.9 % (n = 38) of the cases enrolled in the study, with the parasite prevalence rate of 41.1 % (n = 46). Literacy (OR = 1.9, 95 % CI 0.9–4.3) and CD4 T-cell count <200 (OR = 2.5, 95 % CI 1.1–5.7) were found to be associated with enteric parasite infection. Similarly, CD4 T-cell count <200 was found to be associated with opportunistic parasitic infection (OR = 3.2, 95 % CI 1.2–7.8). Among opportunistic parasites, *Giardia duodenalis* was the most common (28.3 %, n = 13) one. Multi-parasitism was observed in six patients (15.8 %).

**Conclusion:**

Enteric parasitic infections are common in HIV-infected people. The poor immune status as indicated by low CD4 T-cell count may account for higher risk of both opportunistic and non-opportunistic enteric parasitic infection.

## Background

Globally, HIV/AIDS has been a major health problem and the scourge of HIV/AIDS is most unfortunate for resource poor countries like Nepal. In Nepal, more than 25,000 people are living with HIV and only 39 % of them are in reach of life-saving highly active antiretroviral therapy (HAART) [1]. The severity is increased due to opportunistic infections. Enteric parasites are one of the most common infections among HIV/AIDS patients [2–4]. Studies report that several factors including lower CD4 T-cell count, diarrhea, living in a rural area and poor nutrition have been associated with the higher prevalence of enteric parasitic infection in HIV-infected individuals [5–8].

In addition, the occurrence of multi-parasitism has been reported and can lead to negative health outcomes [9, 10] and the condition is worsened in socially and economically marginalized communities [11]. Nepal is among the countries with overlapping high rate of HIV and parasitic infections. Therefore, this study was conducted to detect enteric parasitic infections among HIV-seropositive patients visiting Tribhuvan University Teaching Hospital (TUTH) and association with the immune status of the patients.

## Methods

### Study setting and study population

A cross-sectional type study was carried among the HIV patients attending the TUTH for their regular HAART therapy and HIV counseling from July 2011 to June 2012. Tribhuvan University Teaching Hospital with a total of 444 beds is the largest tertiary care hospital in Nepal that deals with all type of diseases and conditions requiring medical attention.

A total of 112 HIV patients were included in the study and stool specimen was collected from each of them. Patients who were not infected with HIV were excluded from the study.

### Specimen collection, transportation and processing

Fresh stool specimens were collected in labeled, leak proof, clean sterile plastic containers according to the World Health Organization (WHO) standard procedure [12], and transported maintaining cold chain, as quickly as possible to Public Health Research Laboratory, Institute of Medicine for laboratory investigation. Stool specimens were examined microscopically for ova, cysts, oocyst, or parasites, using different techniques. The stool samples were fixed in 10 % formalin, concentrated using formyl/ethyl acetate [13] and Sheather’s sucrose floatation technique and stained in modified acid-fast staining technique [14]. The direct observation was made in saline (0.85 % NaCl solution) and Modified Ziehl Neelsen staining was used for smears of concentrated specimens to examine *Cyclospora cayetanensis, Cryptosporidium* spp. and *Cystoisospora belli* (CCC) [12, 15].

### CD4 T-cell count

The patient’s recent CD4 T-cell count was noted from the record they possessed.

### Statistical analysis

All the results were recorded in the worksheet, entered and analyzed using Statistical Package for the Social Sciences (SPSS) (SPSS Inc., Chicago, version 16). Descriptive statistics were carried out. Frequencies were calculated for categorical variables. Proportions were compared using Fisher’s exact test. Odds ratio (OR) and 95 % confidence intervals (CI) were calculated using a bivariate logistic regression. Tests were considered significant at P value <0.05.

### Ethical approval

The approval for the study was obtained from the Research Department, TUTH. All the participants were explained about the purpose and their participation was voluntary in this study. Informed verbal and written consent was taken from each participant. Moreover, all personal information of the participants was kept confidential. Participants diagnosed as being infected with intestinal parasites were referred appropriately for treatment.

### Operational definitions

A participant was defined as intestinal parasite positive if the stool specimen was positive for at least one of either a pathogenic protozoa or a helminths in microscopic examination. Similarly, a participant was categorized as intestinal parasite negative if the stool specimen on microscopic examination was not positive for pathogenic intestinal parasites. Diarrhea was defined as three or more runny or liquid stool passed within 24 h [16]. Multi-parasitism was defined as the concurrent infestation of a single host individual with two or more parasite species [17].

## Results

A total of 112 stool samples were collected from HIV patients undergoing HAART therapy from TUTH. Out of the samples processed, more than a third patients (33.9 %) were identified with parasites while the prevalence of parasites was 41.1 % (n = 46). The chances of having parasitic infections in females was 1.4 times more than in males. Similarly, illiterate patients were about two times more likely to be infected with parasites than the literate ones (Table 1).

**Table 1:** Factors associated with parasitic infection in HIV patients

Variables	Parasite positive (%)	Odds ratio (95 % CI)	P value
Number of participants (n = 112)	38 (33.9)	–	
Mean age (±SD) (15–64) in years	38.0 ± 10.1	–	
Gender			
Male (n = 65)	20 (30.8)	1	
Female (n = 47)	18 (38.3)	1.4 (0.6–3.1)	0.41
Literacy			
Literate (n = 50)	13 (26.0)	1	
Illiterate (n = 62)	25 (40.3)	1.9 (0.9–4.3)	0.05

### Immune status of cases and enteric parasites prevalence

The chances of parasitic infections in persons with CD4 T-cell count <200 was 2.5 times higher than those with CD4 T-cell count ≥200. Similarly, the chances of getting diarrhea in patients with CD4 T-cell count of <200 was more than two times higher (Table 2).

**Table 2:** Association of parasitic infection and diarrhea with CD4 T-cell count

CD4 T-cell/µL	Total (%)	Parasites detected (%)	OR (95 % CI)	Diarrhea (%)	OR (95 % CI)
<200	30 (26.8)	18 (60.0)	2.5 (1.1–5.7)*	4 (13.3)	2.2 (0.4–9.9)
≥200	82 (73.2)	28 (34.1)	1	3 (3.7)	1
Total (%)	112 (100.0)	46 (41.1)	–	7 (6.3)	–

* Significant difference at P < 0.05

The most frequently identified parasite was *Giardia duodenalis* (syn. *Giardia intestinalis* and *Giardia lamblia*) (28.3 %, n = 13). Altogether, 69.5 % (n = 32) opportunistic parasites: *G. duodenalis,* CCC and *Blastocystis hominis* were identified. The opportunistic parasites were dominantly identified (46.6 %, n = 14) in patients having CD4 T-cell count <200. Similarly, the chances of opportunistic infection in patients having CD4 T-cell count <200 was about 3.5 times higher (OR = 3.4, 95 % CI 1.1–10.1) than in other patients. Non-opportunistic parasite, *Entamoeba histolytica* was identified in higher number in both patients with CD4 T-cell count <200 (13.3 %, n = 4) and CD4 T-cell count ≥200 (8.5 %, n = 7) (Table 3).

**Table 3:** Distribution of parasitic infection in HIV patients

Parasites (n = 46)	Number (%)	CD4 T-cell count		OR (95 % CI)
		<200 (n = 30) (%)	≥200 (n = 82) (%)	
Non-opportunistic				
*E. histolytica*	11 (23.9)	4 (13.3)	7 (8.5)	1.2 (0.4–4.4)
*E. coli*	3 (6.5)	0 (0.0)	3 (3.7)	
Opportunistic				
*G. duodenalis*	13 (28.3)	5 (16.7)	8 (9.8)	3.2 (1.2–7.8)*
*C. cayetanensis*	8 (17.4)	3 (10.0)	5 (6.1)	
*Cryptosporidium* spp.	9 (19.6)	4 (13.3)	5 (6.1)	
*C. belli*	1 (2.2)	1 (3.3)	0 (0.0)	
*B. hominis*	1 (2.2)	1 (3.3)	0 (0.0)	

* Significant difference at P < 0.05

### Multiple parasitisms in HIV patients

Out of 38 patients in whom parasites were detected, six patients (15.8 %) showed multi-parasitism. Two patients (5.3 %) were simultaneously infected with three parasites: *E. histolytica*, *C. cayetanensis*, and *Cryptosporidium* spp. (Table 4).

**Table 4:** Presence of multi-parasitism in HIV patients

Parasites co-infection	Number of patients (n = 38) (%)
*E. histolytica* + *C. cayetanensis* + *Cryptosporidium* spp.	2 (5.3)
*E. histolytica* + *Cryptosporidium* spp.	1 (2.6)
*E. histolytica* + *C. cayetanensis*	1 (2.6)
*E. histolytica* + *E. coli*	1 (2.6)
*C. cayetanensis* + *Cryptosporidium* spp.	1 (2.6)

## Discussion

In the present study, more than a third of the HIV patients undergoing HAART therapy from TUTH were found to have parasitic infections. In different studies done in Nepal, the prevalence of enteric parasites in HIV patients ranged from 22.4 % to more than 35.0 % under different settings [7, 18, 19]. The present study showed that chance of infection with parasites in illiterates was nearly double as compared to the literates. In concordance to our finding, a similar study from India reported lower percentage of parasitic infection owing to a better educational status [20]. Our study also suggested that females had higher chance of parasitic infection than males and this finding is supported by the study done in India [20]. On the contrary, other studies from Nepal [18] and Nigeria [21] showed that more males had parasitic infections than females. The sexual division of labor and the sexual division of responsibility together with local ecological, environmental, economic and cultural factors influence exposure to infection and risk of disease [22]. In developing countries like Nepal, a high disparity exists between male and female in terms of education opportunity, nutritional status and access to medical facilities; the gross effect of which can be accounted for a higher chance of parasitic infection in females than male in current study. However, the role of gender and literacy in parasitic infections is always contradictory and further rigorous research is required.

This study showed that chance of parasitic infections in persons with CD4 T-cell count <200 was 2.5 times higher than those with CD4 T-cell count ≥200. In studies done in Nepal, more than three-fifth [18] to four-fifth [7] of the HIV patients with CD4 T-cell count <200 had parasitic infections. Other studies have depicted the importance of immunity in parasitic infections, [23, 24] which supports our study. The present study showed that there was more than two times higher chances of getting diarrhea in patients with CD4 T-cell count <200. This finding is supported by several studies where higher prevalence of diarrhea was observed in lower CD4 T-cell counts patients [7, 8, 25–27]. The association between diarrhea, lower CD4 T-cell count and presence of intestinal parasites is little understood and needs to be further studied.

In the present study, *G. duodenalis* was the most common parasite (28.3 %, n = 13). In different studies done in Nepal, 6.7–35.0 % of *G. duodenalis* were identified in HIV patients [18, 28, 29]. The non-opportunistic parasites identified were protozoan parasites: *E. histolytica* and *Entamoeba coli,* and no helminthes were identified. However, other studies showed the prevalence of helminthes among HIV patients [7, 21, 30].

In our study, about 7 out of 10 parasites identified were opportunistic parasites: *G. duodenalis,* CCC and *B. hominis*. These parasites have been considered opportunistic by other authors as well [31–33]. *Giardia duodenalis,* was the most common (28.3 %, n = 13) opportunistic parasite identified, which was higher as compared to other studies done in Nepal [7, 18]. In other parts of the world, the prevalence ranged from 9.3 % in India [34] to 43.6 % in Ethiopia [32]. Generally, the variation in the prevalence of cryptosporidial infections could be related to the processing of single stool specimen, which might underestimate the prevalence of cryptosporidial infection [35]. Moreover, oocyst excretion is usually variable [35, 36]. The prevalence of *C. cayetanensis* was 17.4 % (n = 8), which is slightly higher than in other studies done in Nepal [7, 18]. *Cystoisospora belli* and *B. hominis* were less frequently identified parasites in the current study. However, the prevalence of *B. hominis* varied from 1.2 to 6.2 % in studies done in Nepal [7, 18, 28].

The opportunistic parasites were dominantly identified (46.7 %, n = 14) in patients having CD4 T-cell count <200. However, a study done in Nepal showed lower prevalence of opportunistic parasites (27.4 %) [7]. Low CD4 T-cell count is significantly associated with opportunistic parasitic infections as shown by different studies [7, 8, 37, 38]. It has also been established that T lymphocytes and the cytokines they produce play a crucial role in determining the outcome of parasitic infection [39].

We observed multi-parasitism in six patients (15.8 %) in our study. Studies have shown there existed multi-parasitism in Nepal [28] and other parts of the world [33, 40, 41]. A simultaneous infection with different parasites can be a chance, that is, each infection is independent from other infections. In addition, high numbers of parasites and the presence of different species in certain individuals can be due to genetic and immunological predisposition to parasite infections [42, 43].

This study was not without limitations. Firstly, the patients might have treated themselves with anti-parasitic treatments, some of which such as metronidazole, mebendazole and albendazole are easily available without prescription in Nepal. Secondly, single stool sample of the patients was analyzed, which might lead to missed parasitological diagnoses. Thirdly, HIV negative control groups were not included in this study, which might be helpful in comparing different variables among the two groups.

## Conclusion

Enteric parasitic infections still remain significant health problem in Nepal especially among HIV-infected persons with and without diarrhea. 69.5 % of parasites identified were opportunistic in nature among which the majority of the opportunistic parasitic infections were observed in the patients with CD4 T-cell count <200. The current finding also highlights the importance of detection of multi-parasitism among HIV-seropositive patients. It is equally important that the concerned organizations help to manage and improve quality of life of HIV-infected individuals and to avoid morbidity and mortality due to opportunistic pathogens.
